# Natural Killer Cell Dysfunction Is Emerging During Midlife Obesity

**DOI:** 10.1111/acel.70707

**Published:** 2026-09-15

**Authors:** Ruojia Biao, Xudong Wang, Jiantao Fu, Yifan Guo, Junming He, Juan Du

**Affiliations:** ^1^ Peking University Ditan Teaching Hospital Beijing China; ^2^ National Key Laboratory of Intelligent Tracking and Forecasting for Infectious Diseases, Beijing Ditan Hospital Capital Medical University Beijing China; ^3^ Beijing Institute of Infectious Diseases Beijing China; ^4^ National Center for Infectious Diseases, Beijing Ditan Hospital Capital Medical University Beijing China; ^5^ Beijing Key Laboratory of Viral Infectious Diseases, Beijing Ditan Hospital Capital Medical University Beijing China; ^6^ School of Clinical Medicine, Beijing Tsinghua Changgung Hospital Tsinghua University Beijing China; ^7^ Institute for Organ Transplant and Bionic Medicine Tsinghua University Beijing China

**Keywords:** dysfunction, middle age, NK cells, obesity, WAT

## Abstract

Individuals in the middle‐aged demographic are notably more vulnerable to obesity, facing a higher risk of mortality from all causes. However, it remains unclear whether the reduction in life expectancy is related to dysregulated natural killer (NK) cell function triggered by the microenvironment of adiposity in middle age. In the present study, we observed that healthy middle‐aged donors and mice displayed compromised NK cell function compared to their younger counterparts, particularly in males. The quantity and maturation of NK cells were significantly diminished in both spleen and white adipose tissues (WATs), coinciding with an increase in WAT volume and a reduced basal metabolic rate in middle‐aged mice. We further established that heightened apoptosis coupled with a reduction in cell proliferation might contribute to the diminished NK cell counts in the spleens of middle‐aged mice. NK cell function was significantly reduced in the spleen, bone marrow, and adipose tissues of these middle‐aged mice. Young mice with diet‐induced obesity also exhibited impaired NK cell function. In summary, our findings indicate a reduction in both the quantity and functional efficacy of NK cells, which is associated with the rising prevalence of obesity during middle age.

## Graphical abstract

Midlife obesity was associated with impaired NK cell function, including decreased degranulation and IFN‐γ production. The loss of NK cell function might explain the greater mortality risk in middle‐aged individuals, highlighting the importance of monitoring immune alterations during midlife.

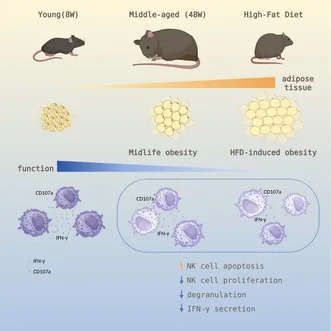

**Abbreviations**
BAT: brown adipose tissue
BM: bone marrow
DCs: dendritic cells
DEGs: differentially expressed genes
eWAT: epididymal white adipose tissue
iWAT: inguinal white adipose tissue
NK cell: natural killer cell
NKT cell: natural killer T cell
Pb: peripheral blood
PBMCs: peripheral blood mononuclear cells
SP: spleen
WAT: white adipose tissue

## 1 Background

Obesity typically emerges during middle age (30–49 years) and progresses into early aging (40–65 years) on a global scale (Flegal et al. 2016; Gallagher et al. 2000; Wang et al. 2021). In mammals, adipose tissue is primarily classified into two types: white adipose tissue (WAT), which is mainly involved in energy storage, and brown adipose tissue (BAT), which is responsible for generating heat to maintain body temperature (Enerbäck et al. 1997; Waldén et al. 2012). WAT is further divided into visceral adipose tissue and subcutaneous adipose tissue (Gesta et al. 2007). From middle age to early aging, humans often experience an accumulation of WAT, particularly in the mass of visceral adipose tissue (Flegal et al. 2010; Vishvanath et al. 2016). The increase in adipose tissue occurs through two primary mechanisms: adipocyte hypertrophy and adipogenesis. Recent studies have demonstrated that, in addition to adipocyte hypertrophy, the adipogenesis of adipose progenitor cells significantly contributes to the expansion of visceral adipose tissue during middle age in mouse models (Wang et al. 2025). Mouse models enable in vivo observation of adipogenesis, revealing that the rate of adipogenesis in young adult mice is notably low, mirroring trends observed in young adult humans. As mice enter middle age, there is a gradual increase in body weight for both males and females, with males experiencing a significantly higher gain compared to females, similar to patterns seen in humans (Flegal et al. 2010). Due to the greater weight increase noted in male mice, studies often focus on males. WAT is the initial organ that exhibits age‐related transcriptomic alterations starting from middle age, positioning WAT as a leading contributor to organismal aging (Franceschi et al. 2018). However, the impact of WAT accumulation during middle age on the immune system remains understudied. WAT is a highly adaptable organ, capable of responding to various metabolic challenges, both physiological and pathological, through alterations in volume and transcriptomic programming (Schaum et al. 2020; Zhou et al. 2020). It is believed that increased adiposity contributes to the acceleration of aging and reduces life expectancy (Blüher et al. 2003). Pathways that promote life‐span—such as sirtuin, forkhead box proteins, and JNKs—typically inhibit adipogenesis and decrease the overall mass of WAT (Armoni et al. 2006; Kanfi et al. 2010; Picard et al. 2004; Roichman et al. 2017; Wang et al. 2005; Wu et al. 2014). However, it remains unclear whether the reduction in life expectancy is related to dysregulated immune responses triggered by the microenvironment of adiposity in middle age.

Emerging epidemiological data suggest that middle‐aged adults suffering from obesity face a notably higher risk of mortality compared to their counterparts with similar health issues in other age groups (Chen et al. 2019). Adipose tissue is frequently recognized as a metabolically active organ capable of modulating immune responses. Chronic low‐grade inflammation, referred to as inflammaging, is another significant characteristic of aging adipose tissue (Franceschi et al. 2018; Zhang et al. 2023). This phenomenon is characterized by elevated levels of proinflammatory cytokines, infiltration of immune cells, and other inflammatory indicators. The infiltration of immune cells to the WAT intensifies with age (Valero‐Muñoz et al. 2016). Adipose tissue macrophages play a crucial role in promoting inflammaging in midlife obesity (Zhou et al. 2025). The changes in other immune cells, particularly natural killer cells in midlife obesity, remain to be thoroughly explored.

In the present study, we found that middle‐aged healthy donors exhibited diminished NK cell function compared to young male donors. Furthermore, we utilized young and midlife male mice to track the immune system and the infiltration of immune cells in adipose tissue in vivo. Our findings revealed that the number and maturation of NK cells were significantly impaired in spleen and WATs of middle‐aged mice, which was accompanied by an accumulation of WATs and a reduced basal metabolic rate. The maturation of NK cells underwent a significant decline in both spleen and adipose tissue, with disrupted activating and inhibitory receptors during midlife obesity. Increased apoptosis and decreased cell proliferation might contribute to the reduced NK cell numbers in spleen and adipose tissues of middle‐aged mice. NK cell function was significantly diminished in spleen, bone marrow, and adipose tissues of middle‐aged mice, indicating a severely compromised NK‐mediated immune response. Specifically, we observed that an increase in obesity in middle age was associated with a decline in both NK cell numbers and their surveillance capabilities.

## 2 Results

### 2.1 Middle‐Aged Healthy Donors Exhibited Diminished Cytotoxic Function of NK Cells Compared to Young Donors in Males

**FIGURE 1:**
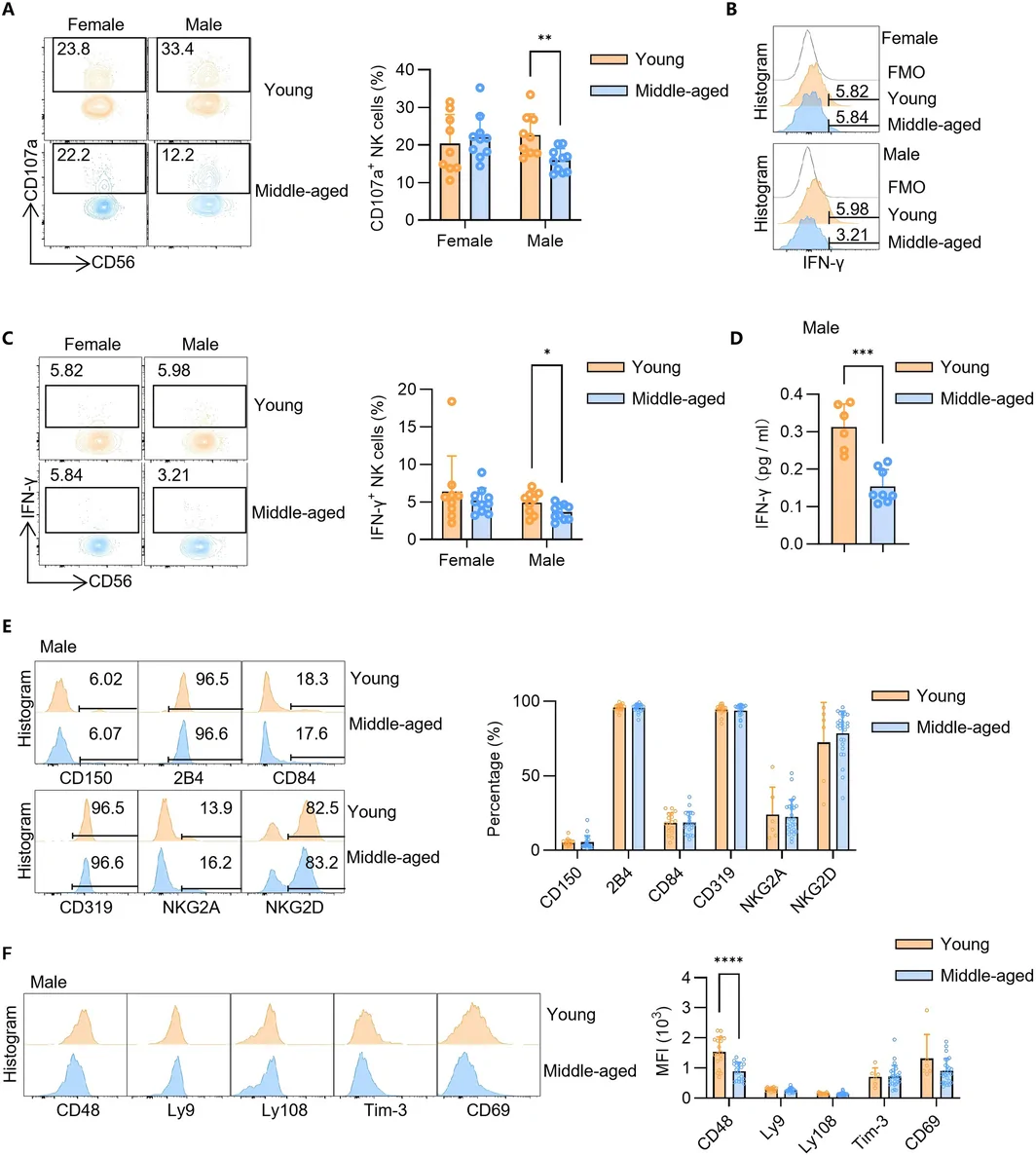
People in middle age display impaired function and responsiveness of NK cells. (A–C) Analysis of NK cell function ex vivo from young (18–40 years old) or middle‐aged (41–65 years old) humans. PBMCs were stimulated with IL‐15, and then CD107a^+^ (A) or IFN‐γ^+^ (B, C) NK cells (CD3^−^CD56^+^) were analyzed by flow cytometry. *n* = 9–10 participants per age group. (B) Representative flow cytometry histograms of IFN‐γ expression in NK cells (CD3^−^CD56^+^), with fluorescence minus one (FMO) controls included. (D) IFN‐γ secretion by human peripheral blood NK cells from young and middle‐aged donors following PMA and ionomycin (P + I) stimulation, as measured by ELISA. Each dot represents one donor. *n* = 6–8 participants per age group. (E, F) Flow cytometric analysis displays the expression of development‐associated receptors on NK cells (CD3^−^CD56^+^) from young (18–40) or middle‐aged (41–65) humans. Cell percentage (E) or Mean Fluorescence Intensity (MFI) (F) is displayed. *n* = 6–26 participants per age group. Each symbol represents an individual person. Data were analyzed using a two‐tailed Student's *t*‐test. The graph represents the mean ± SD, *p < 0.05, **p < 0.01, ***p < 0.001, and ****p < 0.0001.

Among the key roles of NK cells are the expression of CD107a and the release of IFN‐γ, both of which signify their capacity to eliminate target cells and produce cytokines (Vivier et al. 2012). In vitro experiments stimulating NK cells with IL‐15 activate the expression of CD107a and the secretion of IFN‐γ. To investigate whether the function of NK cells is affected during the midlife obesity process in humans, we analyzed the CD107a and IFN‐γ expression of NK cells from young (18–40 years) and middle‐aged (41–65 years) healthy adults (Dallan et al. 2024; Wang et al. 2025). NK cells from female young donors showed comparable CD107a expression levels to those of the female middle‐aged donors, whereas NK cells from male young donors exhibited significantly higher levels of CD107a expression associated with cytotoxic function compared to those of male middle‐aged donors (Figure 1A). The IFN‐γ expression of NK cells in male middle‐aged donors was significantly decreased compared to young male donors, whereas the IFN‐γ expression of young NK cells showed no difference compared to that of middle‐aged donors in the female group (Figure 1B,C). To more directly evaluate NK cell cytokine‐secreting function, IFN‐γ levels in the supernatants of sorted CD3^−^CD56^+^ NK cells stimulated with PMA/ionomycin (P + I) were quantified by ELISA. Consistent with the intracellular cytokine staining results, NK cells from middle‐aged male donors secreted significantly lower amounts of IFN‐γ compared with those from young donors (Figure 1D), indicating an age‐associated decline in NK cell effector function. To determine whether the receptors on the surface of NK cells are affected in the middle‐aged group, we detected the expression of CD150, 2B4, CD84, CD319, NKG2A, NKG2D, CD48, Ly9, Ly108, Tim‐3, and CD69 on NK cells involved in regulating NK cell function in donors. No significant difference was observed in the percentages of CD150, 2B4, CD84, CD319, NKG2A, and NKG2D between young and middle‐aged groups (Figure 1E). Additionally, young donors showed higher expression of CD48 on NK cells than middle‐aged donors. However, the expression of Ly9, Ly108, Tim‐3, and CD69 showed no statistical difference between the two groups (Figure 1F). Thus, the decreased cytotoxic function and cytokine production of NK cells might be characteristic of NK cells in the middle‐aged male group.

### 2.2 Middle‐Aged Mice Exhibit an Accumulation of WATs, Accompanied by a Reduced Basal Metabolic Rate

**FIGURE 2:**
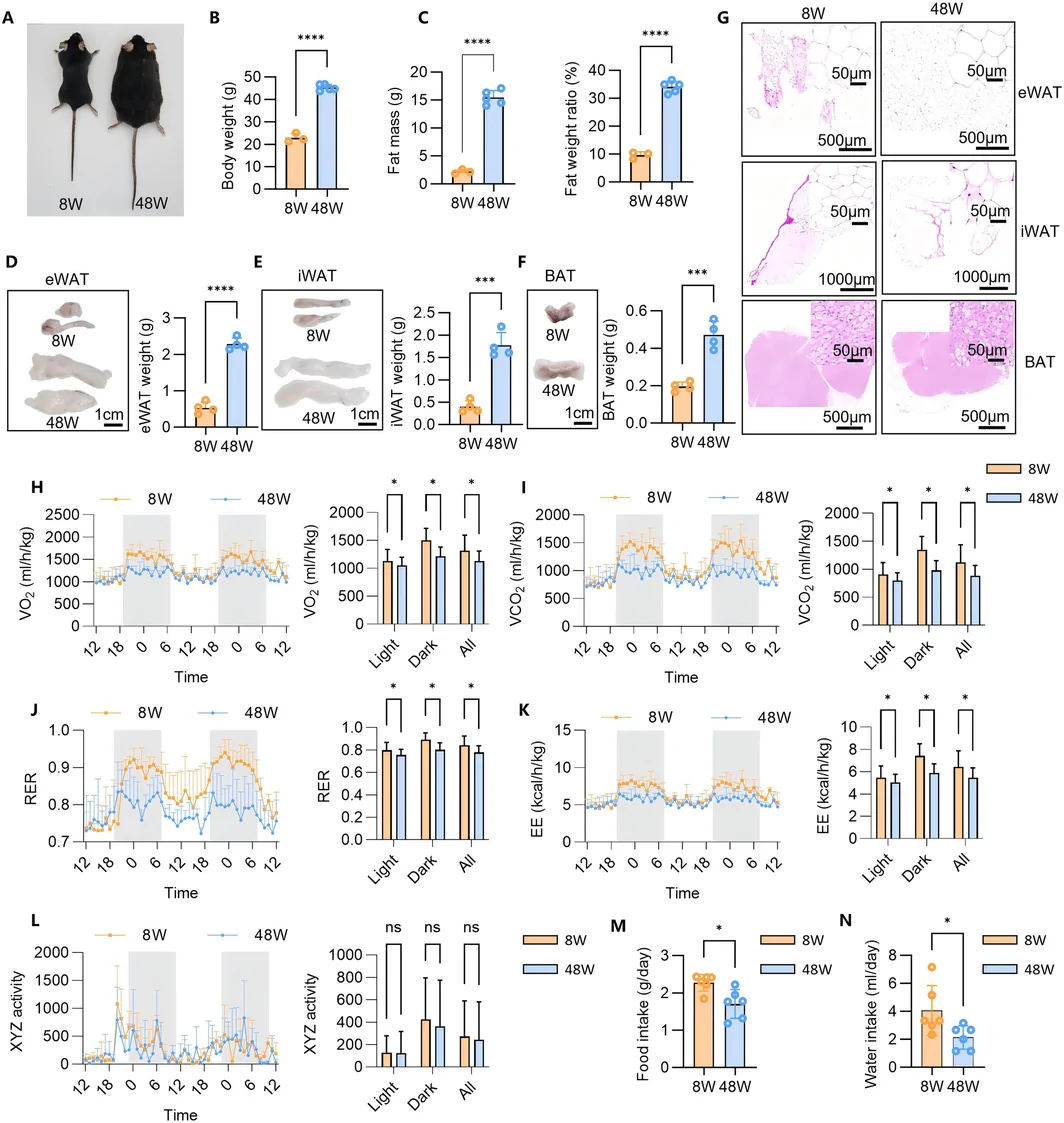
Middle‐aged mice display substantial fat gain and reduced metabolic rate. (A) Images of 8‐week‐old and 48‐week‐old male mice. (B) Body weight of male C57BL/6J mice from 8‐week‐old and 48‐week‐old. *n* = 3–5 mice per age group. (C) Magnetic resonance imaging (MRI) was used to measure the fat mass of 8‐week‐old and 48‐week‐old male mice. *n* = 3–5 mice per age group. (D–F) Tissue weight and whole‐tissue pictures of epididymal white adipose tissue (eWAT) (D), inguinal white adipose tissue (iWAT) (E), and brown adipose tissue (BAT) (F) from 8‐week‐old and 48‐week‐old mice. *n* = 4 mice per age group. (G) Representative H&E‐stained images of eWAT, iWAT, and BAT from 8‐week‐old and 48‐week‐old mice. Consistent results across *n* = 3 animals. (H–L) Metabolic cage studies: Oxygen consumption (H), carbon dioxide production (I), respiratory exchange ratio (J), energy expenditure (K), and activity (L) were determined for 8‐week‐old and 48‐week‐old mice. *n* = 4 mice per age group. Data were analyzed using a two‐tailed Student's *t*‐test corrected by False Discovery Rate (FDR). An asterisk represents a “discovery” (q < Q). (M, N) Food intake (g/day) (M) and water intake (mL/day) (N) were detected. *n* = 6 mice per age group. Each symbol represents an individual mouse. Data were analyzed using a two‐tailed Student's *t*‐test. The graph represents the mean ± SD, *p < 0.05, ***p < 0.001, ****p < 0.0001.

To further elucidate the effects of body metabolism in middle age, we investigated changes in adipose accumulation and metabolism in middle‐aged (48 weeks) and young (8 weeks) male mice. A significantly greater increase in body weight was observed in middle‐aged mice than their younger counterparts (Figure 2A,B). The fat mass and fat weight ratio were significantly elevated in middle‐aged mice (Figure 2C). Among the various fat depots, both epididymal WAT (eWAT) and inguinal WAT (iWAT) showed significant increases in middle‐aged mice relative to young mice (Figure 2D,E). Although brown adipose tissue (BAT) was also increased in midlife, the browning of this tissue was significantly diminished (Figure 2F). In addition to the enlargement of adipose tissue, adipocyte hypertrophy was observed in both eWAT and iWAT (Figure 2G). We subsequently evaluated whole‐body metabolism using metabolic cages. Middle‐aged mice displayed decreased oxygen consumption and carbon dioxide production during both day and night when compared to young mice (Figure 2H,I). We noted a significantly lower respiratory exchange ratio (RER) and whole‐body energy expenditure (EE) in middle‐aged mice compared to the young mice (Figure 2J,K), indicating a substantial reduction in basal metabolism of the middle‐aged mice. The activity levels of middle‐aged mice in XYZ directions were comparable to those of young mice (Figure 2L). Additionally, food and water intake were also reduced in midlife (Figure 2M,N). Therefore, middle‐aged mice demonstrated a pattern of increased adiposity, accompanied by reduced energy expenditure, decreased food intake, and comparable activity levels.

### 2.3 The Number and Maturation of NK Cells Were Impaired in Middle‐Aged Mice

**FIGURE 3:**
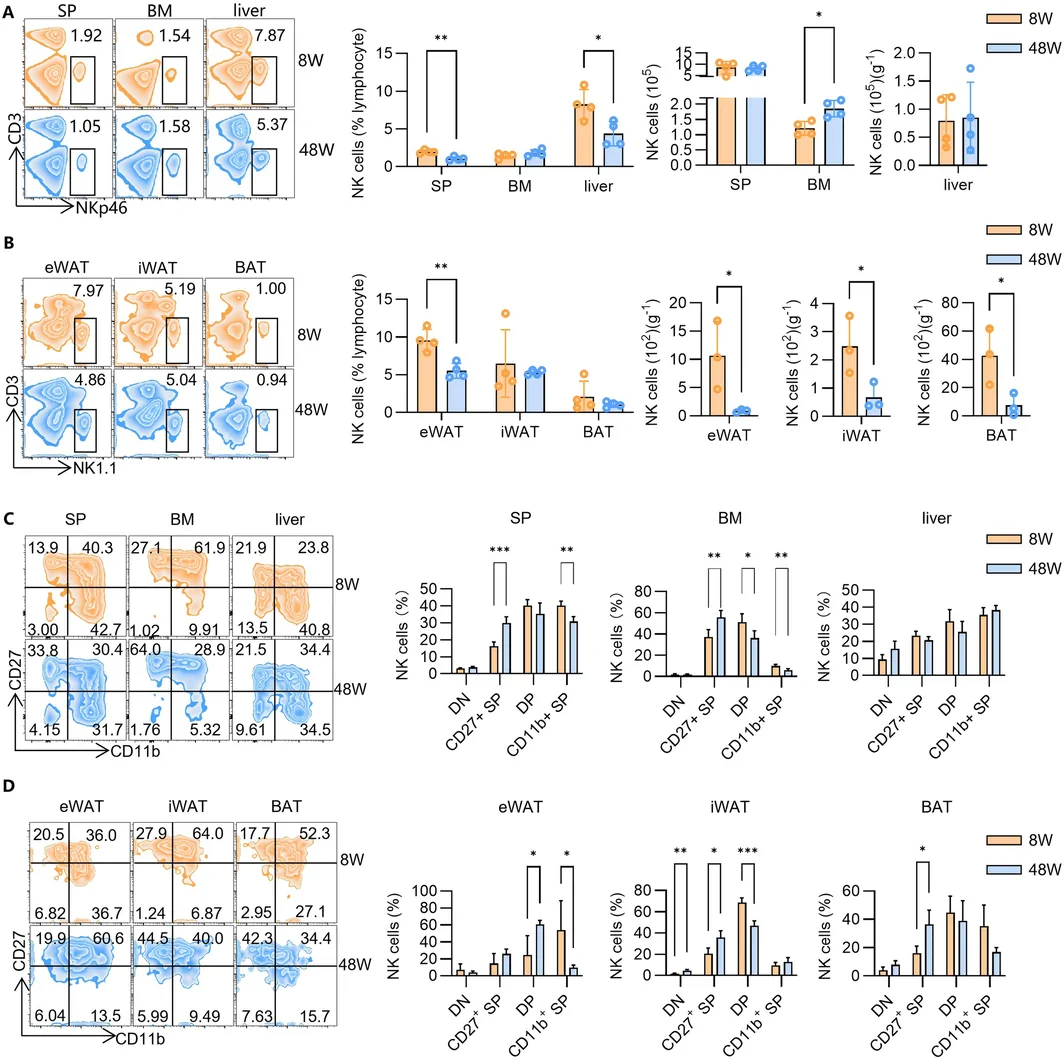
Middle‐aged mice display impaired NK cell development. (A) Flow cytometric analysis of NK cells in the spleen (SP), bone marrow (BM), and liver of 8‐week‐old and 48‐week‐old mice. Representative plots and quantifications of NK cell percentage (CD3^−^NKp46^+^ % lymphocyte) and number are shown. *n* = 4 mice per age group. (B) Flow cytometric analysis of NK cells in the eWAT, iWAT, and BAT of 8‐week‐old and 48‐week‐old mice. Representative plots and quantifications of NK cell percentage (CD3^−^NK1.1^+^ % lymphocyte) and number are shown. *n* = 3–4 mice per age group. (C) Flow cytometric analysis of four subsets of NK cells in the SP, BM, and liver of 8‐week‐old and 48‐week‐old mice. Representative plots and quantifications are shown. DN represents CD27^−^CD11b^−^ NK cells; CD27^+^ SP represents CD27^+^CD11b^−^ NK cells; DP represents CD27^+^CD11b^+^ NK cells; CD11b^+^ SP represents CD27^−^CD11b^+^ NK cells. *n* = 4 mice per age group. (D) Flow cytometric analysis of four subsets of NK cells in the eWAT, iWAT, and BAT from 8‐week‐old and 48‐week‐old mice. *n* = 4 mice per age group. Each symbol represents an individual mouse. Data were analyzed using a two‐tailed Student's *t*‐test. The graph represents the mean ± SD, *p < 0.05, **p < 0.01, and ***p < 0.001.

**FIGURE 4:**
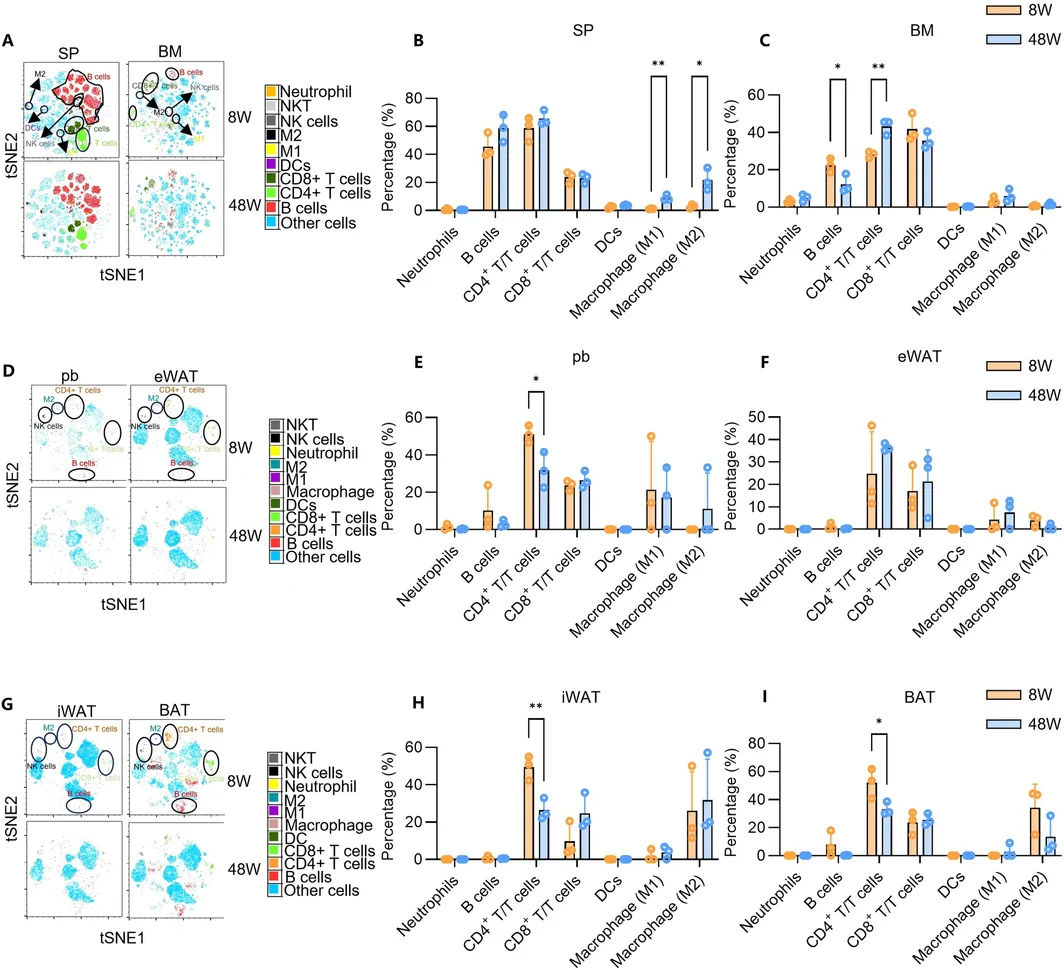
Middle‐aged mice display alterations in immune cell composition. (A) t‐SNE analysis of splenic (SP) and bone marrow (BM) immune cells from 8‐ and 48‐week‐old mice. Single‐cell suspensions from spleen and bone marrow were analyzed by flow cytometry and visualized using t‐distributed stochastic neighbor embedding (t‐SNE). Each dot represents an individual cell, and cells are positioned in the two‐dimensional t‐SNE space based on the similarity of their multiparametric immunophenotypic profiles. Immune cell populations are color‐coded as indicated, and major immune cells are circled, including NK cells, macrophage subsets (M1 and M2), dendritic cells (DCs), CD8^+^ T cells, CD4^+^ T cells, and B cells. Data are shown for mice at 8 weeks (8 W) and 48 weeks (48 W) of age. (B, C) Flow cytometric analysis shows immune cell composition in the spleen (B) and bone marrow (C) of 8‐week‐old and 48‐week‐old mice. Neutrophils, B cells, DCs are expressed as percentages of lymphocytes, whereas CD4^+^/CD8^+^ T cells and M1/M2 macrophages are expressed as proportions of total T cells and macrophages, respectively. *n* = 3 mice per age group. (D) t‐SNE analysis of peripheral blood (pb) and eWAT immune cells from 8‐ and 48‐week‐old mice. Immune cell populations are color‐coded as indicated, and major immune cells are circled. (E, F) Flow cytometric analysis shows immune cell composition in the peripheral blood (E) and eWAT (F) of 8‐week‐old and 48‐week‐old mice. *n* = 3 mice per age group. (G) t‐SNE analysis of iWAT and BAT immune cells from 8‐ and 48‐week‐old mice. Immune cell populations are color‐coded as indicated, and major immune cells are circled. (H, I) Flow cytometric analysis shows immune cell composition in the iWAT (H) and BAT (I) of 8‐week‐old and 48‐week‐old mice. *n* = 3 mice per age group. Each symbol represents an individual mouse. Data were analyzed using a two‐tailed Student's *t*‐test. The graph represents the mean ± SD, *p < 0.05, and **p < 0.01.

To investigate the changes associated with inflammaging during midlife, we examined the immune cell populations in various immune organs, including the primary immune organ bone marrow, secondary immune organ spleen, peripheral blood, liver, and adipose tissues. The relative proportions of NK cell subsets were significantly decreased in the spleen and liver, but not in the bone marrow, in conjunction with midlife obesity (Figure 3A). Additionally, the percentage of NK cells was significantly reduced in eWAT tissue, whereas the percentages in iWAT and BAT remained unaffected in middle‐aged mice (Figure 3B). In middle‐aged mice, NK cell numbers were increased in the bone marrow, whereas no significant changes were observed in the spleen or liver (Figure 3A). In contrast, when normalized to tissue weight, NK cell numbers per gram of tissue were significantly decreased across all three adipose depots (Figure 3B). We quantified the relative proportions of NK cell subsets based on CD27 and CD11b expression (Holmes et al. 2021; Hu et al. 2021; Imianowski et al. 2022). NK cells were categorized into four developmental stages: CD3^−^NK1.1^+^CD27^−^CD11b^−^NK (DN), CD3^−^NK1.1^+^CD27^+^CD11b^−^NK (CD27^+^ SP), CD3^−^NK1.1^+^CD27^+^CD11b^+^NK (DP), and CD3^−^NK1.1^+^CD27^−^CD11b^+^NK (CD11b^+^ SP). The percentage of CD27^+^ SP immature NK cells was increased in the spleen and bone marrow of midlife mice compared to young mice, whereas the proportion of CD11b^+^ SP mature NK cells was decreased in the spleen and bone marrow of midlife mice, similar to the profiles observed in aged mice (72 weeks) (Lu, Li, et al. 2021). This indicated impaired maturation of NK cells in the spleen and bone marrow during middle age. Furthermore, we found that the NK cell maturation pattern in the liver was similar between young and middle‐aged mice, suggesting that middle age did not affect NK cell maturation in the liver as shown in Figure 3C. In adipose tissue, changes in NK cell quantities primarily occurred in WATs. The percentage of DP NK cells was significantly enhanced, whereas the mature CD11b^+^ SP proportion was diminished in the eWAT of middle‐aged mice. In the iWAT of middle‐aged mice, the percentage of DN and CD27^+^ SP immature NK cells increased, whereas the proportion of DP mature NK cells decreased. Notably, BAT tissues from midlife obese mice exhibited a significant increase in the CD27^+^ SP percentage (Figure 3D). To further investigate the differences in other immune cells, we measured the abundances of several common immune cells including neutrophils, NKT, NK, macrophages (M2/M1), DCs, CD8^+^ and CD4^+^ T cells, and B cells to analyze the composition of immune cells using color‐coded t‐SNE overlay plots in the spleen, bone marrow, peripheral blood, and adipose tissues of middle‐aged and young mice (Figure 4A,D,G). Other immune cells, such as neutrophils, B cells, T cells, and DCs, showed no significant differences in the spleen between the two groups. However, M1/M2 macrophages were increased in the spleen of middle‐aged mice (Figure 4B, Figure S1A). In the bone marrow, B cells were decreased and CD4^+^ T cells were increased in the middle‐aged group, whereas macrophages exhibited no significant changes (Figure 4C, Figure S1B). In peripheral blood, only CD4^+^ T cells showed a decreased percentage in middle‐aged mice compared to young mice, contrasting with the trend observed in bone marrow (Figure 4E, Figure S1C). In adipose tissue, no significant differences were found in these immune cells in eWAT, and only CD4^+^ T cells showed a decreased percentage in iWAT and BAT of middle‐aged compared to young mice (Figure 4F,H,I, Figure S1D–F). Further analysis of T cell subsets showed that NKT cells were significantly decreased in the spleen and bone marrow, whereas MAIT cells were increased in eWAT and spleen; and Th2 cells were increased in eWAT and peripheral blood. Th1 and Th17 cells showed no marked changes across the examined tissues (Figure S2). Given that NK cells exhibited the most significant reduction in the spleen, liver, and adipose tissue, our subsequent experiments focused on NK cells during midlife. Overall, during the onset of middle‐aged obesity, the number and maturation of NK cells were significantly reduced in the spleen and WATs.

### 2.4 The Surface Receptors of NK Cells Were Disrupted During Midlife Obesity

**FIGURE 5:**
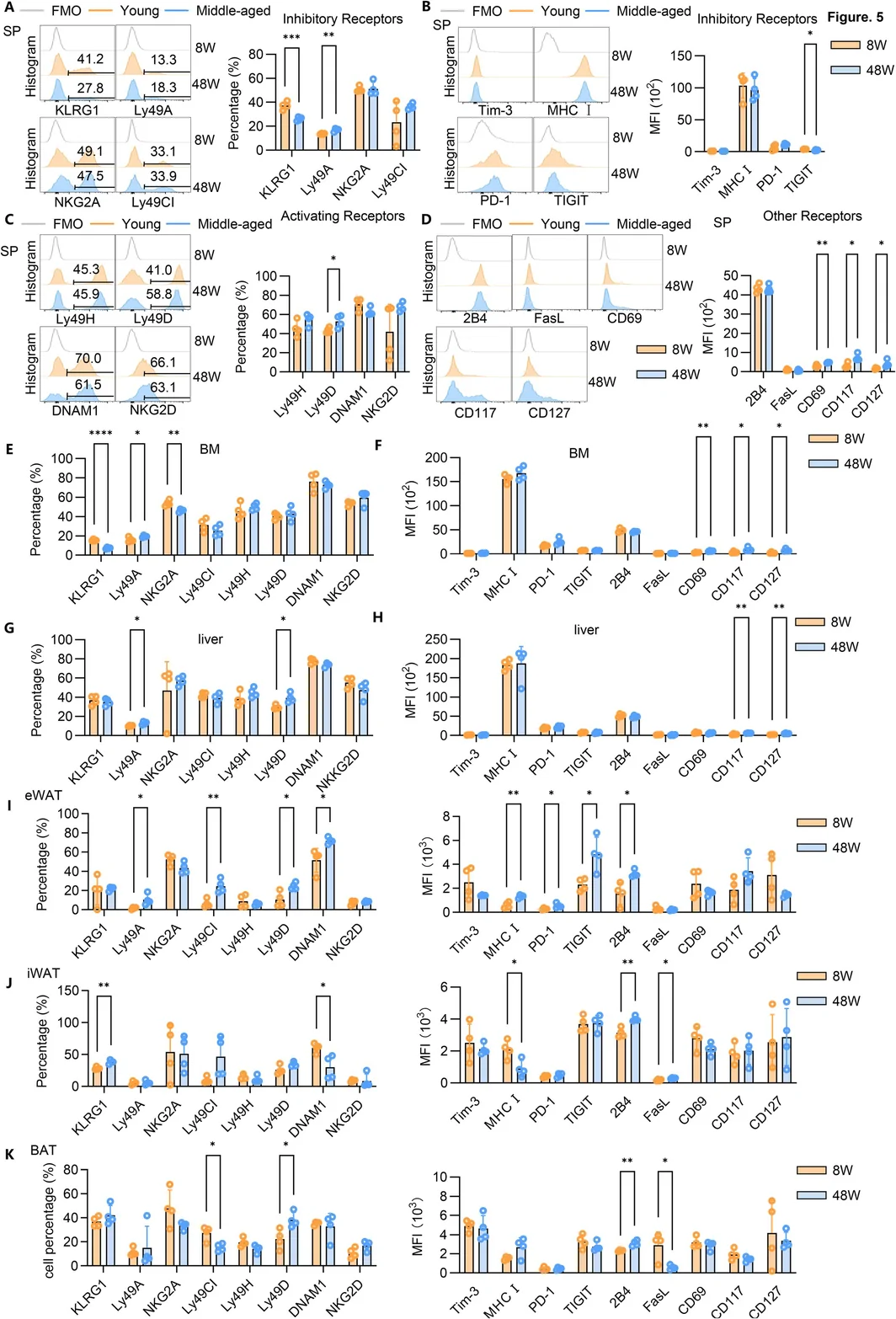
Middle‐aged mice display phenotypic alterations of NK cells. (A–D) Flow cytometric analysis displays the expression of inhibitory receptors (A, B), activating receptors (C), and other development‐associated markers (D) of splenic NK cells (CD3^−^NKp46^+^) from 8‐week‐old and 48‐week‐old mice. Representative histograms and quantification of these markers are displayed. *n* = 4 mice per age group. (E–H) Flow cytometric analysis displays the expression of development‐associated markers on NK cells (CD3^−^NKp46^+^) from BM (E, F) and liver (G, H) from 8‐week‐old and 48‐week‐old mice. Quantification of these markers is displayed. *n* = 4 mice per age group. (I–K) Flow cytometric analysis displays the expression of development‐associated markers on NK cells (CD3^−^NK1.1^+^) from eWAT (I), iWAT (J), and BAT (K) from 8‐week‐old and 48‐week‐old mice. Quantification of these markers is displayed. *n* = 4 mice per age group. Each symbol represents an individual mouse. Data were analyzed using a two‐tailed Student's *t*‐test. The graph represents the mean ± SD, *p < 0.05, **p < 0.01, ***p < 0.001, and ****p < 0.0001.

The function of NK cells is regulated by inhibitory and activating receptors, as well as other receptors expressed on their surface. In the spleen, the inhibitory receptors KLRG1 and TIGIT were significantly downregulated, whereas Ly49A was dramatically upregulated in middle‐aged mice. The levels of other inhibitory receptors, including NKG2A, Ly49CI, Tim‐3, MHC‐I, and PD‐1, remained unchanged in the spleen of midlife mice (Figure 5A,B). In addition, middle‐aged mice displayed a significant increase in the expression of the activating receptor Ly49D, whereas the levels of the other activating receptors, including Ly49H, DNAM1, and NKG2D, were not significantly altered in the spleen (Figure 5C). The expression levels of the activation marker CD69 on NK cells were increased in the spleen of middle‐aged mice. Furthermore, expression levels of the immature markers CD117 and CD127 on splenic NK cells were markedly higher in midlife obese mice. The expression levels of other receptors, such as 2B4 and FasL, associated with NK cell function, were comparable between the spleen of middle‐aged and young mice (Figure 5D). In the bone marrow, KLRG1 and NKG2A were significantly downregulated, whereas Ly49A, CD69, CD117, and CD127 were slightly upregulated in middle‐aged mice (Figure 5E,F). In the liver, Ly49A, Ly49D, CD117, and CD127 were significantly increased in mice with midlife obesity (Figure 5G,H). In eWAT, the expression of Ly49A, Ly49CI, Ly49D, DNAM1, MHC I, PD‐1, TIGIT, and 2B4 was increased in middle‐aged mice (Figure 5I). KLRG1, 2B4, and FasL were upregulated, whereas DNAM1 and MHC I were downregulated in iWAT in middle‐aged mice (Figure 5J). In BAT, Ly49D and 2B4 showed an increase in middle‐aged mice compared to young mice, whereas Ly49CI and FasL showed a decrease in middle‐aged mice (Figure 5K). In summary, the maturation of NK cells was significantly diminished in the spleen and adipose tissue of middle‐aged mice, with increased expression of Ly49D and CD69 on NK subsets and decreased inhibitory receptors KLRG1 and TIGIT in the spleen.

### 2.5 NK Cells Exhibited a Heightened Propensity for Apoptosis and Limited Proliferation in Spleen of Middle‐Aged Mice

**FIGURE 6:**
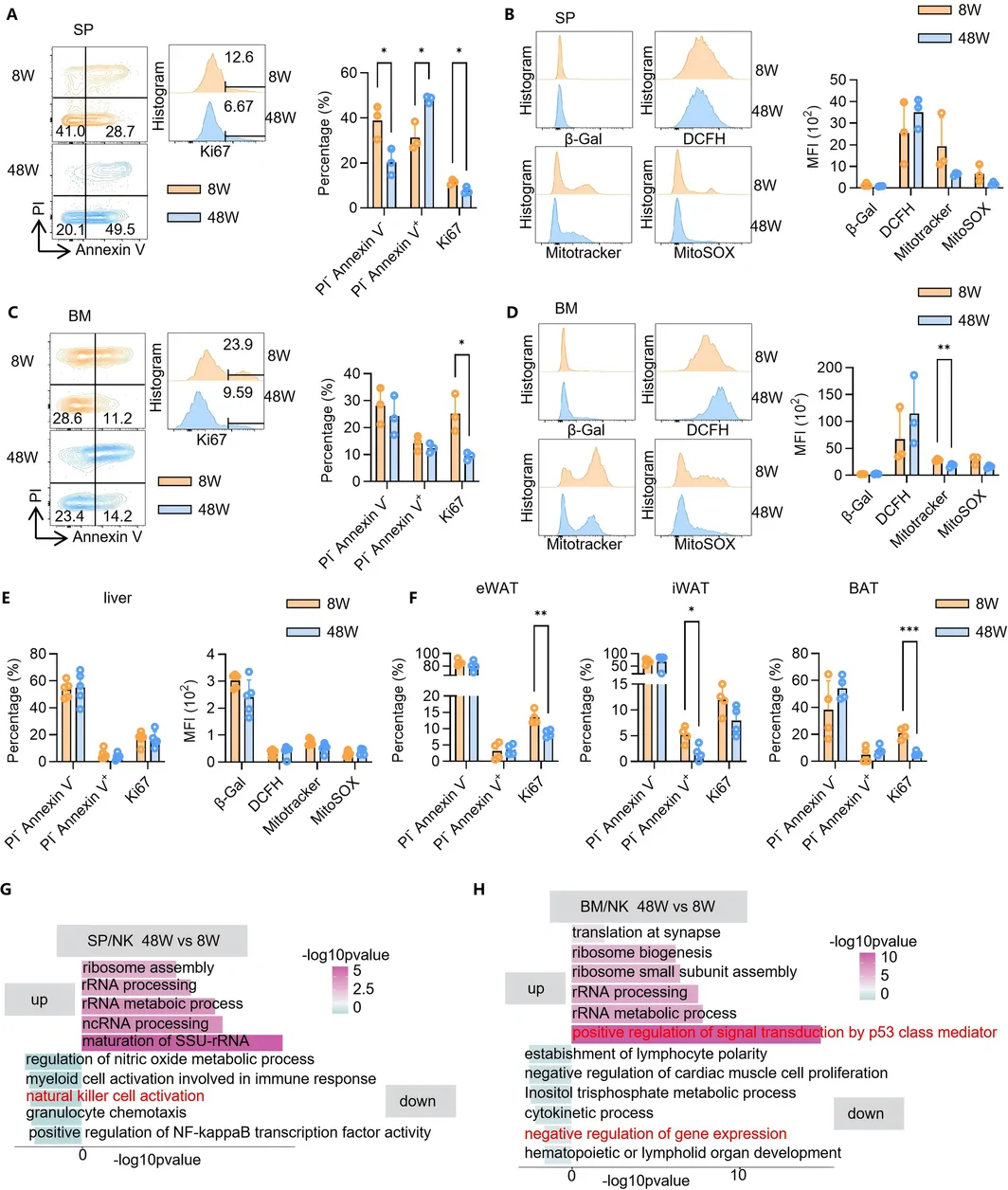
Middle‐aged mice display impaired viability and proliferation of NK cells. (A, C) Flow cytometric analysis of the expression of PI, Annexin V, and Ki67 on splenic (A) and BM (C) NK cells from 8‐week‐old and 48‐week‐old mice. Representative plots and quantification of PI^−^Annexin V^−^, PI^−^Annexin V^+^, and Ki67^+^ NK cells (CD3^−^NKp46^+^) are displayed. *n* = 3 mice per age group. (B, D) Flow cytometric analysis of the senescence (β‐Gal), mitochondrial content (Mitotracker), ROS (MitoSOX), and cellular ROS (DCFH) of splenic (B) and BM (D) NK cells (CD3^−^NKp46^+^) from the 8‐week‐old and 48‐week‐old mice. *n* = 3 mice per age group. (E) Flow cytometric analysis of the expression of PI, Annexin V, Ki67, Mitotracker, MitoSOX, and DCFH on liver NK cells (CD3^−^NKp46^+^) from the 8‐week‐old and 48‐week‐old mice. *n* = 5 mice per age group. (F) Flow cytometric analysis of the expression of PI, Annexin V, and Ki67 on eWAT, iWAT, and BAT NK cells (CD3^−^NK1.1^+^) from the 8‐week‐old and 48‐week‐old mice. *n* = 4 mice per age group. (G, H) Up‐ and down‐regulated pathways of splenic (G) and BM (H) NK cells from 48‐week‐old mice compared with 8‐week‐old mice, based on single‐cell RNA sequencing data. Each symbol represents an individual mouse. Data were analyzed using a two‐tailed Student's *t*‐test. The graph represents the mean ± SD, *p < 0.05, **p < 0.01, and ***p < 0.001.

To determine whether the observed reduction in NK cell numbers was attributable to altered proliferation or survival in middle‐aged mice, we assessed apoptosis rates using Annexin V and Ki‐67 expression. The percentage of PI^−^ Annexin V^+^ NK cells was significantly elevated, whereas their proliferation and the percentage of viable (PI^−^ Annexin V‐) NK cells were markedly reduced in the spleen of middle‐aged mice compared to their younger counterparts (Figure 6A). Notably, there were no significant differences in senescence marker β‐gal or cytosolic ROS marker DCFH between the two groups in the spleen. Additionally, fluorescent indicators for mitochondrial mass (MitoTracker) and ROS‐generating mitochondria (MitoSOX) exhibited a decreasing trend, but no statistically significant differences were observed in the spleen between middle‐aged and young mice (Figure 6B). In bone marrow, middle‐aged and young mice showed comparable rates of NK cell apoptosis, whereas significant differences in NK cell proliferation were evident (Figure 6C). Similar to the spleen, no significant differences in senescence β‐gal or cytosolic ROS levels were found between the two groups in bone marrow. MitoTracker levels were significantly diminished in the bone marrow of middle‐aged mice compared to young mice, whereas MitoSOX levels showed a decreasing trend without significant differences (Figure 6D). In the liver, no significant differences were observed between middle‐aged and young mice in NK cell apoptosis, proliferation, senescence β‐gal, mitochondrial mass, mROS or cROS levels (Figure 6E). However, NK cell proliferation was significantly reduced in eWAT and BAT, whereas NK cell apoptosis was decreased in iWAT (Figure 6F). Overall, NK cells with midlife obesity exhibited increased susceptibility to apoptosis in the spleen and reduced proliferation potential in the spleen, BM, eWAT and BAT. To further elucidate the signaling characteristics of NK cells in middle‐aged versus young control mice, we conducted single‐cell RNA sequencing of NK cells isolated from the spleen and bone marrow. Our findings revealed a significant downregulation of signaling pathways associated with NK cell activation in the spleen of middle‐aged mice compared to young mice (Figure 6G). Furthermore, we observed an upregulation of p53 class mediator signaling and a downregulation of negative regulation of gene expression, suggesting that midlife obesity might adversely impact NK cell function (Figure 6H).

### 2.6 Midlife Obesity Severely Compromised NK‐Mediated Surveillance

**FIGURE 7:**
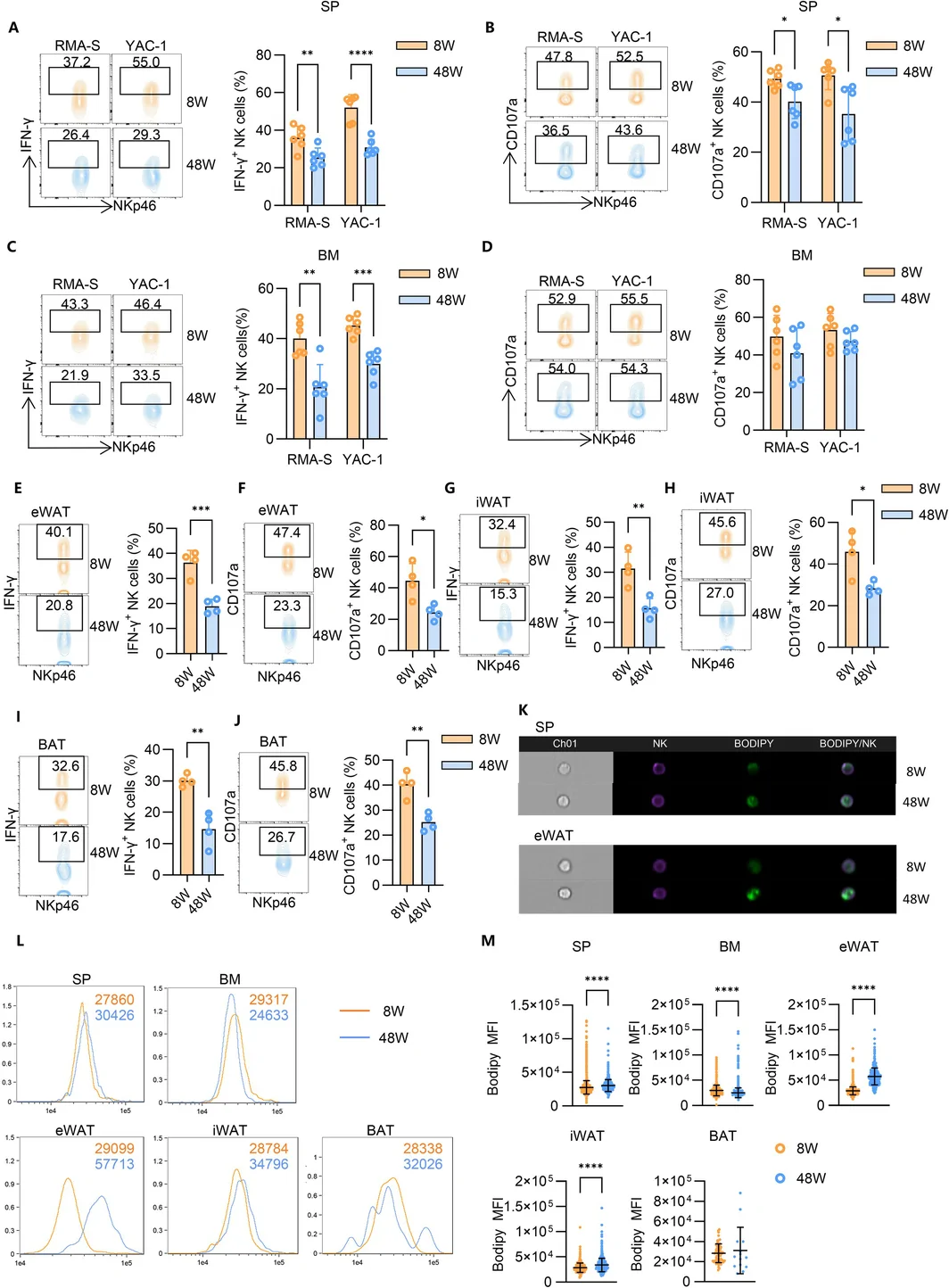
Middle‐aged mice display impaired function of NK cells. (A, B) Analysis of NK cell function ex vivo. Splenocytes were stimulated as indicated (RMA‐S and YAC‐1), and then IFN‐γ^+^ (A) or CD107a^+^ (B) NK cells were analyzed by flow cytometry. *n* = 6 mice per age group. (C, D) Cells from BM were stimulated as indicated (RMA‐S and YAC‐1), and then IFN‐γ^+^ (C) or CD107a^+^ (D) NK cells were analyzed by flow cytometry. *n* = 6 mice per age group. (E–J) Cells from eWAT (E, F), iWAT (G, H), and BAT (I, J) were stimulated by YAC‐1, and then IFN‐γ^+^ (E, G, I) or CD107a^+^ (F, H, J) NK cells were analyzed by flow cytometry. *n* = 4 mice per age group. (K) Representative image of NK cells (CD3^−^NK1.1^+^) stained with Bodipy 493/503 obtained from imaging flow cytometry. NK cells (purple) and Bodipy 493/503 (green). (L) Histograms of Bodipy 493/503 in NK cells (CD3^−^NK1.1^+^) from 8‐week‐old and 48‐week‐old mice, as measured by imaging flow cytometry. MFI values are indicated in the plots. (M) Statistical analysis of Bodipy 493/503 fluorescence intensity in NK cells from different tissues of 8‐week‐old and 48‐week‐old mice, as measured by imaging flow cytometry. Each symbol represents an individual mouse. Data were analyzed using a two‐tailed Student's *t*‐test. The graph represents the mean ± SD, *p < 0.05, **p < 0.01, ***p < 0.001, and ****p < 0.0001.

The primary functions of NK cells, which include the expression of CD107a and the production of IFN‐γ, reflect their capability to eliminate target cells and secrete cytokines. To investigate the function of NK cells in middle‐aged mice, we measured the expression of CD107a and the secretion of IFN‐γ ex vivo with MHC‐1 deficient target tumor cells (YAC‐1 or RMA‐S). The proportion of NK cells from spleen expressing IFN‐γ and surface‐associated CD107a upon stimulation with target cells was significantly reduced in middle‐aged mice compared to young mice (Figure 7A,B). In bone marrow, the percentage of IFN‐γ^+^ NK cells was significantly decreased in middle‐aged mice. However, the proportion of CD107a^+^ NK cells was comparable between the two groups (Figure 7C,D). Since the most significant change observed in middle‐aged mice was the accumulation of adipose tissue, we also examined the function of NK cells within the adipose tissue microenvironment, which plays a crucial role in adipose tissue inflammation. Due to the limited number of NK cells infiltrating the adipose tissue, we only stimulated NK cell function using the MHC‐1 deficient target tumor YAC‐1. In white adipose tissues, including eWAT and iWAT, the proportion of NK cells expressing IFN‐γ and CD107a in response to YAC‐1 was significantly diminished in middle‐aged mice compared to young mice (Figure 7E–H). Even in BAT, the percentage of IFN‐γ^+^ and CD107a^+^ NK cells was significantly lower in middle‐aged than young mice (Figure 7I,J). To further determine whether NK cell dysfunction was accompanied by altered lipid metabolism, intracellular lipid accumulation in NK cells was assessed using Bodipy 493/503 staining. NK cells from middle‐aged mice showed increased lipid accumulation compared with those from young mice across spleen, eWAT and iWAT, with the most pronounced increase observed in eWAT NK cells (Figure 7K–M). However, lipid accumulation in bone marrow NK cells showed a decreasing tendency (Figure 7L,M). In conclusion, these data further demonstrate that NK cell function is significantly diminished in spleen, bone marrow, and adipose tissues of middle‐aged mice, which may be partly associated with increased lipid accumulation.

### 2.7 Obesity Induced by a High‐Fat Diet Also Impairs NK Cell Function in Young Mice

**FIGURE 8:**
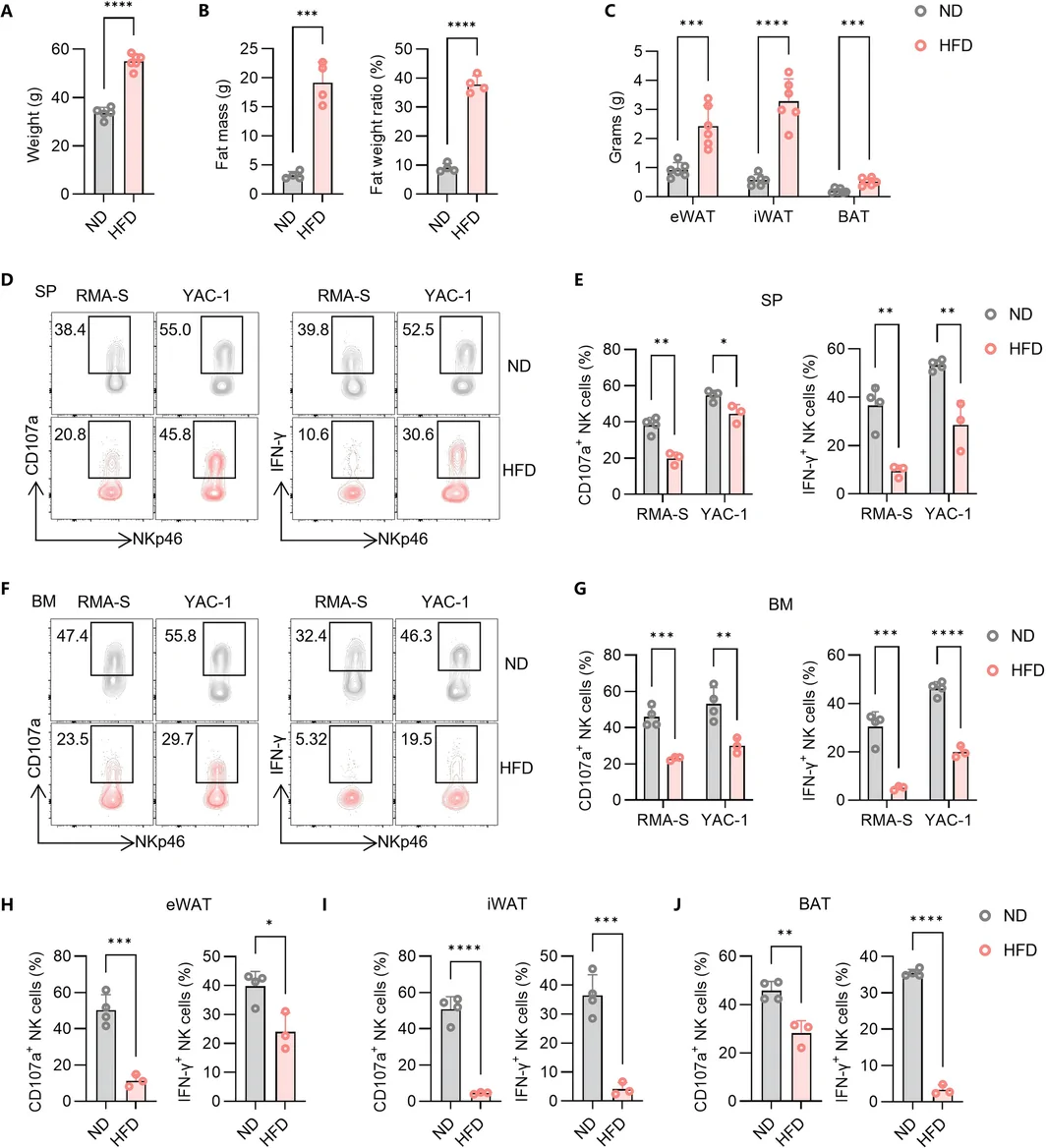
Mice fed a high‐fat diet exhibit impaired NK cell function. (A) Body weight of male C57BL/6J mice from ND and HFD mice. *n* = 5–6 mice per group. (B) Magnetic resonance imaging (MRI) was used to measure the fat mass of ND and HFD mice. *n* = 4 mice per group. (C) Tissue weight of eWAT, iWAT, and BAT from ND and HFD mice. *n* = 6 mice per group. (D, E) Analysis of NK cell function ex vivo. Splenocytes were stimulated as indicated (RMA‐S and YAC‐1), and then IFN‐γ^+^ or CD107a^+^ NK cells were analyzed by flow cytometry. *n* = 3–4 mice per group. (F, G) Cells from BM were stimulated as indicated (RMA‐S and YAC‐1), and then IFN‐γ^+^ or CD107a^+^ NK cells were analyzed by flow cytometry. *n* = 3–4 mice per group. (H–J) Cells from eWAT (H), iWAT (I), or BAT (J) were stimulated by YAC‐1, and then IFN‐γ^+^ or CD107a^+^ NK cells were analyzed by flow cytometry. *n* = 3–4 mice per group. Each symbol represents an individual mouse. Data were analyzed using a two‐tailed Student's *t*‐test. The graph represents the mean ± SD, *p < 0.05, **p < 0.01, ***p < 0.001, and ****p < 0.0001.

To isolate the effects of adiposity from aging‐related changes, we employed a high‐fat diet (HFD)‐induced obesity model to examine the impact of obesity on NK cell function. Eight‐week‐old male mice were fed either a high‐fat diet (HFD; 60% kcal from fat, Research Diets D12492) or a normal chow diet (ND; 10% kcal from fat) for 16 weeks. Compared to ND mice, HFD mice exhibited a significant increase in body weight (Figure 8A), accompanied by elevated total fat mass and fat weight ratio (Figure 8B). Consistent increases were observed across multiple adipose depots, including eWAT, iWAT, and BAT (Figure 8C). NK cell function was subsequently evaluated through stimulation with the MHC‐I–deficient target cells RMA‐S and YAC‐1. Similarly, splenic NK cells from HFD mice exhibited a significantly reduced percentages of IFN‐γ^+^ and CD107a^+^ cells compared with those from ND controls (Figure 8D,E). A similar reduction in the proportions of IFN‐γ^+^ and CD107a^+^ NK cells was also observed in the bone marrow of HFD mice (Figure 8F,G). Given the pronounced expansion of adipose tissue in HFD mice and our previous observations of age‐associated NK cell dysfunction in adipose tissue, we further examined NK cell function within adipose depots following YAC‐1 stimulation. Consistently, NK cells isolated from eWAT, iWAT, and BAT displayed markedly reduced degranulation and IFN‐γ production in HFD mice compared with ND mice (Figure 8H–J). Collectively, these findings indicate that diet‐induced obesity is associated with a broad impairment of NK cell function across lymphoid organs and adipose tissue.

## 3 Discussion

NK cells are cytotoxic lymphocytes that serve as a primary line of defense in the human immune system by recognizing and eliminating tumor cells and virally infected cells. Aging is recognized to impact the immune system, resulting in a gradual decline in the functionality of immune cells and alterations in the distribution of immune cell subsets within the bloodstream. Recent research has indicated that macrophages in adipose tissue can activate midlife obesity in response to a high‐fat diet (Zhou et al. 2025). NK cells are also important mediators of obesity‐associated inflammation. Short‐term HFD‐induced (2–3 weeks) obesity has been reported to activate NK cells and promote IFN‐γ‐mediated inflammation and insulin resistance, whereas sustained lipid overload may progressively compromise NK‐cell effector function. In 8‐week‐old Apoc3TG mice, splenic NK cells exhibited reduced IFN‐γ production and impaired degranulation. Similarly, in long‐term HFD‐fed mice (≥ 8 weeks), splenic NK cells showed downregulation of cytotoxicity, whereas peripheral blood NK cells displayed decreased IFN‐γ production (Hu et al. 2021; Michelet et al. 2018; Wensveen et al. 2015). However, it remains uncertain whether middle‐age obesity modifies the phenotype and function of other immune cells, particularly NK cells. In this article, we demonstrated that decreased cytotoxic function and cytokine production of NK cells were the characteristic of NK cells in middle‐aged men. Furthermore, we investigated NK cells in spleen, bone marrow and adipose tissue of young and midlife mice in vivo. Our results indicated a significant impairment in both the number and maturation of NK cells in the spleen and WATs, which were accompanied by an increase in WAT accumulation and a reduction in basal metabolic rate in middle‐aged mice. The maturation process of NK cells showed a noticeable decline in the spleen and adipose tissue, with disruptions observed in both the activating and inhibitory receptors of NK cells during midlife obesity. An increase in apoptosis coupled with a decrease in cell proliferation may contribute to the lower NK cell counts in the spleen and adipose tissues of middle‐aged mice. Furthermore, NK cell function was substantially reduced in the spleen, bone marrow, and adipose tissues of these mice, highlighting a significant compromise in NK‐mediated activity. Notably, we observed that the rise in obesity during midlife is linked to a reduction in NK cell numbers and their surveillance capabilities. The loss of NK cell quantity and function might explain the greater mortality risk in middle‐aged individuals.

White adipose tissue is a highly plastic organ that responds to physiological and pathological metabolic challenges by undergoing changes in size and gene expression. In the absence of metabolic stress, healthy young mice and humans exhibit low rates of adipocyte turnover. Recent findings indicated that the adipogenesis of adipose progenitor cells significantly contributed to adipose tissue expansion during middle age (Wang et al. 2025). A significant conclusion from this research was that, despite the generally low turnover rate of adipocytes in younger adults, adipogenesis became activated in middle age. The expansion of adipose tissue during this period resulted from both increased adipocyte hypertrophy and high adipogenic capacity of adipose progenitor cells. Starting from middle age, adipose tissue was the first organ to display transcriptomic changes associated with aging, positioning it as a major factor in the aging process of the organism (Schaum et al. 2020; Zhou et al. 2020). Due to excessive accumulation of adipose tissue in midlife, both middle‐aged mice and humans tend to exhibit reduced oxygen consumption, lower energy expenditure, and diminished basal metabolic rates compared to their younger counterparts (Huang et al. 2022; Sakers et al. 2022; Xie et al. 2023). A defining feature of aging in adipose tissue is the presence of chronic low‐grade inflammation, characterized by elevated levels of proinflammatory cytokines and various inflammatory markers, which can occur even in the absence of infection or injury. Adipose tissue macrophages, the predominant immune cell subpopulation in white adipose tissue, were crucial in promoting inflammaging (Garg et al. 2014; Sawaki et al. 2023; Thevaranjan et al. 2017; Zhou et al. 2025). However, the impact of midlife metabolic conditions on the functionality of other immune cell types remains poorly understood. Reports indicated that humans and mice with obesity exhibited both numerical and functional impairments in NK cells, leading to increased vulnerability to cancer and infections (Michelet et al. 2018). Given that obesity has been recognized to affect NK function, we hypothesized the potential involvement of NK cells in midlife obesity. Thus, we investigated the role of NK cell phenotype and function using samples from both healthy donors and mice. Our findings demonstrated that the rise in obesity during midlife was associated with a reduction in NK cell numbers and their surveillance capabilities. The increase in adipose tissue during midlife might impair NK cell function through metabolic alterations. These data suggest that adipose tissue‐derived factors contribute to phenotypic and functional changes in NK cells. The cytotoxic capacity of NK cells has been reported to be compromised by intracellular accumulation of lipid droplets (Michelet et al. 2018). Human adipose tissue is also rich in lipid droplets (Obert et al. 2017), suggesting that adipose expansion during middle age may create a lipid‐enriched microenvironment that affects NK cell function. Consistent with this possibility, our results showed increased lipid accumulation in NK cells from middle‐aged mice, with the most pronounced increase observed in eWAT NK cells. These findings suggest that the reduced NK cell function observed in middle‐aged obesity may be influenced by lipid accumulation. Splenic NK cells mainly reflect systemic immune surveillance (Flommersfeld et al. 2021; Kaneko et al. 2024; Michel et al. 2012; Mujal et al. 2025), whereas adipose NK cells are more significantly influenced by the local metabolic microenvironment and contribute to obesity‐associated inflammation (Boulenouar et al. 2017; De Barra et al. 2023; Lee et al. 2016). This difference may result from the different effects of their tissue environments. The spleen supports NK cell activation and systemic immune surveillance, whereas adipose tissue links NK cells more closely to metabolic inflammation. In the context of middle‐aged obesity, which is the focus of our study, increased adiposity is a defining characteristic. Therefore, examining NK cells from both adipose tissue and spleen allowed us to assess local NK cell alterations within the obese metabolic microenvironment as well as systemic NK cell dysfunction associated with middle‐aged obesity.

However, despite previous reports showing age‐associated accumulation of immune cells in adipose tissue, including T cells and B cells (Carey et al. 2024; Lumeng et al. 2011), as well as imbalances in macrophage polarization reflected by altered M1/M2 ratios (Carey et al. 2024; Lu, Huang, et al. 2021), we did not observe comparably pronounced changes in these immune cell populations in our study. This discrepancy is likely attributable to differences in the ages of the animal models examined. Whereas most prior studies of immunological aging focused on substantially older mice (18–22 months old), our study examined middle‐aged mice (~12 months old). Accordingly, the immune microenvironment of adipose tissue during middle age may differ fundamentally from that observed in late‐life aging. As aging progresses, both intrinsic aging of adipose tissue and age‐related immune cell dysfunction are expected to further increase the complexity of the adipose immune microenvironment. Consistent with this notion, immune cells within adipose tissue—including macrophages, B cells, and T cells—may not yet have undergone the extensive remodeling typically observed during advanced aging at 48 weeks of age (~12 months).

In middle age, both humans and mice experience greater weight gain in males than in females (Flegal et al. 2010; Kumar et al. 2008; Wang et al. 2025). Consistent with this observation, we found that the function of NK cells in middle‐aged men was significantly reduced compared to that in younger men, whereas no such difference was observed between these two age groups in women. Male mice exhibited substantial body weight gain during middle age due to an increase in white adipose tissue mass, whereas female mice only demonstrated moderate weight gain (Wang et al. 2025). Furthermore, the enhancement of adipogenesis and the emergence of the age‐enriched adipose progenitor cells occurred primarily in the male visceral adipose tissue. Therefore, in our study, we primarily focused on male participants and male mice for further analyses. We observed that the function of NK cells in middle‐aged male mice was significantly diminished in the spleen, bone marrow, and adipose tissue compared to their younger counterparts. Reports have also indicated that inflammatory signals promote adipogenesis (Lackey and Olefsky 2016; Hotamisligil 2017; Wernstedt Asterholm et al. 2014). The reduced NK cell function might influence the microenvironment of adipogenesis and the activity of other immune cells. Immune cell infiltration in the WAT increased with age, but the infiltration and function of NK cells in WAT remained unclear. Our findings revealed that NK cell infiltration and function in adipose tissues were significantly decreased in middle‐aged mice. Overall, the impaired function of NK cells in both humans and mice during middle age was found to be sex‐specific.

Because the middle‐aged obesity model does not allow clear separation of the effects of aging and obesity on NK cell dysfunction, we examined high‐fat diet‐induced obesity in young mice to assess the impact of adiposity in the absence of aging. In this context, obesity was associated with impaired NK cell function across multiple tissues, suggesting that excess adiposity itself may contribute to the NK cell dysfunction observed during middle age. Consistently, dietary restriction has been reported to enhance NK cell function (He et al. 2024). Although advanced aging independently compromises NK cell cytotoxicity and cytokine production in both humans and mice (Brauning et al. 2022; Lu, Li, et al. 2021; Solana et al. 2014), these effects have been mainly described in elderly populations, whereas the immunological consequences of middle age remain less well defined. Collectively, our findings suggest that, in middle‐aged obesity, obesity may exert a contributory role in NK cell dysfunction. Moreover, NK cell dysfunction was more pronounced in HFD mice than in middle‐aged mice. This may be attributed to the fact that HFD mice exhibited greater adipose tissue accumulation.

Of course, we acknowledge several limitations of the present study. First, the inference regarding differences between middle‐aged obesity and HFD‐induced obesity is indirect and does not fully distinguish middle‐aged obesity from other obesity models. Second, although this study primarily focused on male participants and male mice, we recognize that sex‐specific differences represent an important biological variable that was not addressed here. These limitations highlight the need for future studies to further refine obesity models and to investigate sex‐dependent differences in NK cell function in greater depth.

In summary, middle‐aged healthy donors and mice exhibited weakened NK cell function compared to younger donors and mice, particularly in males. The number and maturation of NK cells were significantly impaired in spleen and WATs, coinciding with the accumulation of WAT and a reduced basal metabolic rate in middle‐aged mice. We further demonstrated that increased apoptosis and decreased cell proliferation might contribute to the reduced NK cell numbers in the spleen of middle‐aged mice. NK cell function was significantly diminished in the spleen, bone marrow, and adipose tissues of these mice. Moreover, NK cell function was reduced in the HFD‐induced young obesity model. Overall, our findings indicate a decline in NK cell number and surveillance, which is associated with increased obesity in middle age.

## 4 Materials and Methods

### 4.1 Mice

All the mice were C57BL/6 background and maintained under specific pathogen‐free animal facilities. Ethical approval for all experimental procedures involving mice was obtained from the Animal Ethics Committee of Institute of Genetics and Developmental Biology, Chinese Academy of Sciences (AP2023046). Only male mice were used in this study. 8‐ and 48‐week‐old male mice were sacrificed and tissues including spleen, bone marrow, liver, peripheral blood, and adipose tissue were obtained. For histological examination, epididymal white adipose tissue (eWAT), inguinal white adipose tissue (iWAT), and brown adipose tissue (BAT) were fixed in 4% paraformaldehyde, embedded in paraffin, sectioned, stained with H&E, and scanned by light microscopy for metastatic foci. Photomicrographs of H&E‐stained slides of adipose tissue metastases were analyzed with the Image J software (Bethesda, MD, USA).

### 4.2 Participant

A cohort of 122 healthy participants was recruited, including 19 females and 103 males. Donors in all groups were stratified according to age as young (18–40 years) or middle‐aged (41–65 years). Ethical approval for all experimental procedures was approved by the Committee of Ethics at Beijing Ditan Hospital, Capital Medical University in Beijing with informed consent acquired from all participants (No. DTEC‐KY2024‐098‐02). Exclusion criteria were pregnancy, individuals below 18 years old, severe hepatic or renal impairment, opportunistic infections, and other concomitant diseases such as autoimmune disease, malignancy, and cardiovascular diseases.

### 4.3 Isolation of Peripheral Blood Mononuclear Cells (PBMCs)

Peripheral blood samples were isolated using standard Ficoll‐Paque gradient centrifugation according to the instructions of the manufacturer (Amersham Pharmacia Biotech, Sweden).

### 4.4 Imaging Flow Cytometry

Cells were first stained with surface antibodies CD3 and NK1.1 for 30 min, followed by staining with Bodipy 493/503 (Invitrogen, D3922) for 30 min. NK cells and Bodipy expression were acquired at 40× magnification on an Amnis ImageStream^X^ Mk II and analyzed using IDEAS 6.2 software (both Merck Millipore, Billerica, MA, USA).

### 4.5 Flow Cytometry

Cells from different tissues from the indicated mice were resuspended in PBS buffer containing 2% fetal bovine serum and were incubated with surface antibodies for 30 min. PBMCs from participants were incubated with directly conjugated antibodies for 30 min. Then, the cells were fixed and permeabilized using Cytofix/Cytoperm reagent (BD Biosciences). The intracellular antibodies were stained and analyzed by flow cytometry. Monoclonal antibodies include anti‐mouse CD3e (eBio500A2, 145‐2C11), NK1.1 (PK136), CD19 (1D3), CD11b (M1/70), CD27 (LG.7F9), NKp46 (29A1.4), CD4 (GK1.5), CD8 (53‐6.7), F4/80 (BM8), CD11c (N418), Ly6G (1A8), Ly6C (HK1.4), MHC II (M5/114.15.2), KLRG1 (2F1), Ly49A (A1 (Ly49A)), NKG2A (16a11), Ly49CI (5E6), Tim‐3 (RMT3‐23), MHC I (28–14‐8), PD‐1 (29F.1A12), TIGIT (MBSA43), Ly49H (3D10), Ly49D (4E5), DNAM‐1 (10E5), NKG2D (CX5), 2B4 (eBio244F4), FasL (MFL3), CD69 (H1.2F3), CD117 (2B8), CD127 (A7R34), Ki67 (SolA15), CD45 (I3/2.3), CD45R (RA3‐6B2), CD44 (IM7), CCR4 (2G12), CCR6 (29‐2 L17), CXCR3 (CXCR3‐173), CD1d complex (L363), Annexin V, β‐Gal, DCFH, Mitotracker, MitoSOX, and isotype controls were purchased from eBioscience (San Diego, CA), Biolegend (San Diego, CA), or BD Biosciences (Mississauga, Ontario, Canada). Anti‐human antibodies include CD3 (UCHT1); CD14 (M5E2); CD16 (3G8); CD62P (AK4); SLAMF1 (A12); SLAMF2 (TU145); 7‐AAD‐Percp (BD Biosciences, San Diego, CA, USA), CD19 (SJ25C1); CD41 (HIP8); CD56 (HCD56); NKG2A (S19004C); NKG2D (1D11); SLAMF4 (C1.7); SLAMF5 (CD84.1.21); SLAMF7 (162.1); SLAMF3 (HLy9.25); SLAMF6 (hSF6.4.20); Tim‐3 (F38‐2E2); CD69 (FN50). Samples are detected using a flow cytometer (BD FACSymphony A5 Flow Cytometer). The expression level was presented as relative ratio or mean fluorescence intensity (MFI), which was determined by subtracting the mean fluorescence intensity of the isotype control.

### 4.6 IFN‐γ ELISA Measurement

Peripheral blood mononuclear cells (PBMCs) were isolated from human peripheral blood by density gradient centrifugation. NK cells were subsequently purified by flow cytometric cell sorting as CD3^−^CD56^+^ cells. Sorted NK cells were resuspended in complete medium and seeded in 96‐well plates at a density of 1 × 10^5^ cells per well in a total volume of 200 μL. Cells were stimulated with PMA and ionomycin (*P* + I) and incubated for 5 h at 37°C in a humidified incubator with 5% CO_2_. After stimulation, plates were centrifuged at 2000 rpm for 5 min, and culture supernatants were collected for analysis. The concentration of IFN‐γ in the supernatants was measured using a human IFN‐γ high‐sensitivity ELISA kit (LAIZEE BIOTECH, LEH803‐2HS, 96T) according to the manufacturer's instructions.

### 4.7 In Vitro NK‐Cell Function Assays

8‐ and 48‐week‐old mice (C57BL/6J) were intraperitoneally injected with PolyI:C (10 μg/kg), then cells from spleen, BM, and adipose tissue were collected and co‐cultured with NK‐sensitive tumor cells YAC‐1 and RMA‐S in the presence of anti‐CD107a (eBio1D4B). PBMCs were co‐cultured with IL‐15 in the presence of anti‐CD107a (H4A3). After co‐culturing with GolgiStop (BD, Biosciences) for 6 h, cells were harvested and stained with the indicated antibodies, fixed, permeabilized, and then stained with an anti‐IFN‐γ (4S.B3) (mice) antibody or an anti‐IFN‐γ (B27) (human) antibody.

### 4.8 Body Composition Detection

Whole‐body composition of mice is detected by Small Animal Body Composition Analyzer (EchoMRI 3in1).

### 4.9 Metabolic Analysis

Animals aged 8 and 48 weeks were analyzed for oxygen consumption, carbon dioxide production, energy expenditure, respiratory exchange rate, spontaneous locomotor activity, food intake, and water intake using the Small Animal Metabolic Monitoring System (TSE Phenomaster). Animals were maintained in individual cages following a 12/12 h light/dark cycle with water and food supplied ad libitum, and under controlled room temperature (21°C–23°C) and 50% humidity. Metabolic data were collected for 2 days following a 1‐day acclimatation period. Data were measured and collected every 6 min for each experimental mouse. After normalization to body weight, all time‐point measurements from individual mice within each group were included in the analysis. Data were subsequently analyzed using a two‐tailed Student's *t*‐test corrected by False Discovery Rate (FDR). An asterisk presents a “discovery” (q < Q). Data are expressed as mean ± SD.

### 4.10 Single‐Cell Isolation

Mice were euthanized by cervical dislocation, and bone marrow (BM) cells were isolated by crushing the bones from Femur. BM cells were then passed through MACS SmartStrainer (70 μm, Cat# 130‐098‐462; MiltenyiBiotec) and resuspended in PBS buffer. Spleens were mashed through MACS SmartStrainer with a syringe plunger and rinsed with PBS to prepare single‐cell suspensions. The re‐suspended cells were treated with a 10‐fold volume of 1× Red Blood Cell Lysis Solution (Cat# 130‐094‐183; MiltenyiBiotec). The viability, concentration, and clumping rate of the cells were determined with Acridine Orange/Propidium Iodide (AOPI) stain agent (Cat# F23001; Logos Biosystems) on LUNA‐FLTM Automated Fluorescence Cell Counter (Cat# L20001; Logos Biosystems). Before applying to the next procedure, the samples were centrifuged and re‐suspended with an adequate volume of 1× phosphate‐buffered saline (PBS) + 0.04% BSA + 1 U/μL RNase inhibitor to get a final concentration of 700–1200 cells/μL.

### 4.11 Single‐Cell Capture and cDNA Library Construction

The samples were prepared using the Single Cell 3′ Kit v3.1 and Chip G on Chromium Single Cell Controller (10× Genomics) and ETC811 Thermal cycler (EASTWIN) according to the manufacturer's instruction. Briefly, the appropriate volume of nuclease‐free water, Master Mix, and single‐cell suspension for targeting 10,000 single cells were mixed and added to Chip G to generate the gel bead‐in‐emulsions (GEMs) with Gel Beads and Partitioning Oil on Chromium Single Cell Controller. The GEMs were dispensed into the tube strip on ice and sent for reverse transcription within 1 h. The products were then added with Recovery Agent to break the single‐cell droplets, and the first‐strand cDNA was isolated and cleaned with Dynabeads Cleanup Mix. Afterwards, the resulting samples underwent steps of cDNA amplification, fragmentation, adaptor ligation and sample index PCR to generate the DNA libraries, with a cleanup procedure using SPRIselect Reagent Kit at the end of each step. Finally, the libraries were assessed with Qseq400 (BiOptic, Taiwan, China) to ensure their quality could meet the requirements for sequencing.

### 4.12 scRNA‐Seq Analysis

Data normalization, batch correction, clustering and visualization of single‐cell datasets are all done by R package Seurat (v4.1.0). We applied a criterion to filter out cells with nFeature RNA less than 200 and more than 2500. To remove low‐quality cells and likely multiplet captures, which is a major concern in microdroplet‐based experiments, we further discarded low‐quality cells where > 15% of the counts belonged to mitochondrial genes. Top variable genes across single cells were identified using the method described in Macosko et al. (2015). The most variable genes were selected using FindVariableGenes function (mean.function = FastExpMean, dispersion.function = FastLogVMR) in Seurat (Butler et al. 2018). Differentially expressed genes (DEGs) were identified using the FindMarkers function (test.use = presto) in Seurat (Butler et al. 2018). *p* value < 0.05 and |log_2_foldchange| > 0.5 was set as the threshold for significantly differential expression. KEGG analyses were performed by clusterProfiler (Xu et al. 2024). *p*‐value was adjusted by using Benjamini–Hochberg correction.

### 4.13 T‐Distributed Stochastic Neighbor Embedding (t‐SNE) Analysis

Single cells were isolated, stained, and tested with flow cytometry (BD FACSymphony A5 Flow Cytometer). Flow cytometry data were analyzed with FlowJo software and R language (Version 4.0.5). CD45‐positive cells were exported as an expression matrix based on the channel values of all samples in the FlowJo software. Data from selected cells were merged into one matrix, normalized by channel, and analyzed with the t‐SNE algorithm by the Seurat package (version 4.0.2). The parameters used for t‐SNE dimensionality reduction analysis were CD3, NK1.1, CD11b, CD27, CD19, CD4, CD8, F4/80, Ly‐6G/Ly‐6C, MHC‐II, and CD11c.

### 4.14 Statistics

The data presented in this article represent the mean ± SD. A two‐tailed Student's *t*‐test was performed. A *p* value of less than 0.05 was considered significant. Statistical significance was indicated by *p < 0.05, **p < 0.01, ***p < 0.001, and ****p < 0.0001, respectively. All statistical analyses were performed using GraphPad Prism 10.4.0 software.

## Author Contributions

R.B. performed the experiments and analyzed the data. X.W., J.F., and Y.G. performed the experiments. J.H. participated in the critical review of the manuscript and revised the manuscript. J.D. designed the experiments, analyzed the data, and wrote the manuscript. All authors contributed to the article and approved the submitted version.

## Funding

This work was supported by National Natural Science Foundation of China (82371565 to J.D.), and the Beijing Tsinghua Changgung Hospital Fund (No. 2025QMC010, 12026C01007 to J.H.).

## Ethics Statement

Ethical approval for all experimental procedures involving mice was obtained from the Animal Ethics Committee of Institute of Genetics and Developmental Biology, Chinese Academy of Sciences (AP2023046). Ethical approval for all experimental procedures involving humans was approved by the Committee of Ethics at Beijing Ditan Hospital, Capital Medical University in Beijing with informed consent acquired from all participants (No. DTEC‐KY2024‐098‐02). Confidentiality and privacy were assured. The study complied with the Declaration of Helsinki.

## Conflicts of Interest

The authors declare no conflicts of interest.

## Supporting information


**Figure S1:** Middle‐aged mice display changes in immune cell composition. (A) Flow cytometric analysis shows immune cell composition in the spleen of 8‐week‐old and 48‐week‐old mice. Neutrophils, B cells, T cells, macrophages, and DCs are expressed as percentages of lymphocytes, whereas CD4^+^/CD8^+^ T cells and M1/M2 macrophages are expressed as proportions of total T cells and macrophages, respectively. *n* = 3 mice per age group. (B–F) Flow cytometric analysis shows immune cell composition in the BM (B), peripheral blood (pb) (C), iWAT (D), BAT (E), and eWAT (F) from 8‐week‐old and 48‐week‐old mice. *n* = 3 mice per age group. Each symbol represents an individual mouse. Data were analyzed using a two‐tailed Student's *t*‐test. The graph represents the mean ± SD, *p < 0.05 and **p < 0.01. Comparisons where *p* > 0.05 (not statistically significant) are labeled as “ns”.

**Figure S2:** Middle‐aged mice display alterations in T cell subsets. (A) Flow cytometric analysis shows T cell subsets in the spleen of 8‐week‐old and 48‐week‐old mice. NKT cells, Th1/2/17 and MAIT cells are expressed as percentages of lymphocytes. *n* = 3 mice per age group. (B–F) Flow cytometric analysis shows T cell subsets in the BM (B), peripheral blood (C) and adipose tissues (D–F) of 8‐week‐old and 48‐week‐old mice. *n* = 3 mice per age group. Each symbol represents an individual mouse. Data were analyzed using a two‐tailed Student's *t*‐test. The graph represents the mean ± SD, *p < 0.05, **p < 0.01 and ***p < 0.001.

## Data Availability

All data needed to evaluate the conclusions in the paper are present in the paper or in the public database.
